# Analysis of Growth Trajectories and Verification of Related SNPs in *Populus deltoides*

**DOI:** 10.3390/ijms242216192

**Published:** 2023-11-10

**Authors:** Yaolin Wang, Zesen Wang, Sheng Zhu, Huixin Pan, Changjun Ding, Meng Xu

**Affiliations:** 1 State Key Laboratory of Tree Genetics and Breeding, Co-Innovation Center for Sustainable Forestry in Southern China, Nanjing Forestry University, Nanjing 210037, China; wangyaolin@njfu.edu.cn (Y.W.); zesen@njfu.edu.cn (Z.W.); zhusheng@njfu.edu.cn (S.Z.); hxpan@njfu.com.cn (H.P.); 2 State Key Laboratory of Tree Genetics and Breeding, Key Laboratory of Tree Breeding and Cultivation of State Forestry Administration, Research Institute of Forestry, Chinese Academy of Forestry, Beijing 100091, China

**Keywords:** *Populus deltoides*, SNPs, growth model, longitudinal growth trait, growth trajectory

## Abstract

As an important timber genus with high economic and ecological values, *Populus* is a model for dissecting the genetic architecture of growth traits in perennial forest trees. However, the genetic mechanisms of longitudinal growth traits in poplar remain incompletely understood. In this study, we conducted longitudinal genetic analysis of height and diameter at breast height (DBH) in eleven-year poplar clones using ultra-deep sequencing datasets. We compared four S-shaped growth models, including asymptotic, Gompertz, logistic, and Richard, on eleven-year height and DBH records in terms of five metrics. We constructed the best-fitting growth model (Richard) and determined poplar ontogenetic stages by virtue of growth curve fitting and likelihood ratio testing. This study provides some scientific clues for temporal variation of longitudinal growth traits in *Populus* species.

## 1. Introduction

Section *Aigeiros* Duby (*Populus*), including *P. deltoides*, *P. nigra*, and their interspecific hybrids, has significant economic and genetic significance [1]. Among the representatives of *Aigeiros*, *Populus deltoides* is known for its rapid growth rate, strong resistance to various environmental conditions, and wide adaptability. These characteristics make *P. deltoides* suitable for various applications such as plywood, lumber, paper production, woody biomass, and bioenergy production [2]. Complex growth traits are highly correlated with wood and biomass yields of poplar. Compared to herbaceous plants such as *Arabidopsis thaliana*, poplars, as perennial woody species for bioenergy production, present characteristics such as long generation times, huge body-size, and high heterozygosity that impede the study of the mechanisms of inheritance of complex traits [3]. Therefore, elucidating the genetic basis of complex traits in growth trajectories is an important aspect of poplar breeding research. Ontogenetic growth models, such as logistic, Richard, and Gompertz models, have been widely adopted to exhibit the long-term patterns of physiological and growth traits in perennial woody plants, including poplar [4,5,6]. These models can provide valuable insights into the genetic control of growth and dynamic traits in poplar.

Quantitative trait locus (QTL) mapping is a well-established technique for studying complex traits and identifying functional genes with those traits [7]. It has been widely applied in various plant species, including *Zea mays* [7], *Oryza sativa* [8], *Populus* [9], *Cunninghamia lanceolate* [10], and *Eucommia ulmoides* [11]. Genome-wide association studies GWASs) have indeed emerged as a powerful tool for dissecting QTLs and identifying genes associated with complex traits. GWASs have been extensively used for many tree species, such as *Populus* [12], *Salix suchowensis* [13], *Hevea brasiliensis* [14], and *Camellia sinensis* [15]. These GWASs have significantly contributed to genetic improvement research on tree species.

The growth of forest trees is a complex process, encompassing stages from juvenility to adulthood, and from vegetative growth to reproductive growth. The growth traits of these trees are governed by the combined effects of numerous small effector genes and their interactions. However, current association analysis studies have primarily concentrated on examining specific time points or periods during the growth of forest trees. Analyzing growth traits at individual time points may overlook important functional genes that are involved in the overall growth process. This is especially notable in the case of perennial forest trees, where analyzing dynamic growth traits becomes essential. Researchers have recognized this issue and have conducted numerous studies to investigate the genetic mechanisms underlying the growth of forest trees [16,17,18,19]. The functional mapping approach proposed by Wu and Lin [20] has been widely used for dissecting the genetic architecture of growth traits in forest trees [5,21,22]. By incorporating a temporal dimension, the functional mapping approach takes into account the dynamic nature of growth and captures the genetic effects on traits at different developmental stages. Little is known about the continuous contribution of genetic variant loci to growth-related traits in perennial woody plants during the whole development cycle. Xu et al. performed a growth-related GWAS on *P. deltoides* cv. I-69, *P. × euramericana* cv. I-45, and their 64 F1 progenies and identified trait-associated single-nucleotide polymorphism (SNP) loci [19]. We verified the genotype of twenty GWAS trait-associated loci in twenty-four unrelated *P. deltoides* clones by using resequencing datasets of an average 240× coverage, and we fitted the long-term growth trajectory function of growth traits (e.g., height and diameter at breast height (DBH)) in 11-year-old poplar trees.

## 2. Results

### 2.1. Phenotypic Variation of Growth Traits in 24 P. deltoides Clones 

**Figure 1:**
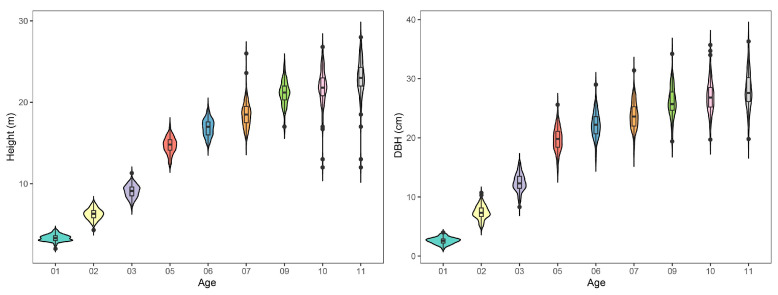
The phenotype distributions of height and diameter at breast height (DBH) displayed in violin plots across different years.

One-way analysis of variance (ANOVA) indicated that significant variations in DBH and height were detected among the poplar clones over the 11-year period (Table S1, *p* < 0.01). Based on the correlation analysis conducted for each year, it has been found that height exhibits a significant correlation with final growth starting from the fifth year and DBH from the second year (Table S2). The range of variation in DBH and height increased as the stand grew, and the phenotypic distribution is displayed in Figure 1. On average, the height of clones was 22.96 m with a range from 12.00 m to 28.00 m, and DBH was 28.03 cm with a range from 19.8 cm to 36.3 cm (Table S3). The S3244 clone exhibited the highest growth performance among the other clones, with a height of 27.17 m and DBH of 35.1 cm. When compared to the control clone NL895, the DBH and height of S3244 were 14.88% and 20.62% higher.

### 2.2. SNP Identification Using Sequencing Data

A total of 24 *P. deltoides* clones were successfully sequenced using the Illumina MiSeq sequencing platform. Following quality control steps, a total of 166,897 clean reads were obtained. The GC content of these reads ranged from 40.35% to 46.82%, indicating that the samples were free from contamination and that the second-generation sequencing technology did not introduce significant bias to the data. Furthermore, the percentage of Q20 bases in each sample was found to be greater than 89.86%, and the content of Q30 bases was higher than 86.8% (Table S4). The above indicators validate the usability of the obtained clean data for further analysis and interpretation.

**Figure 2:**
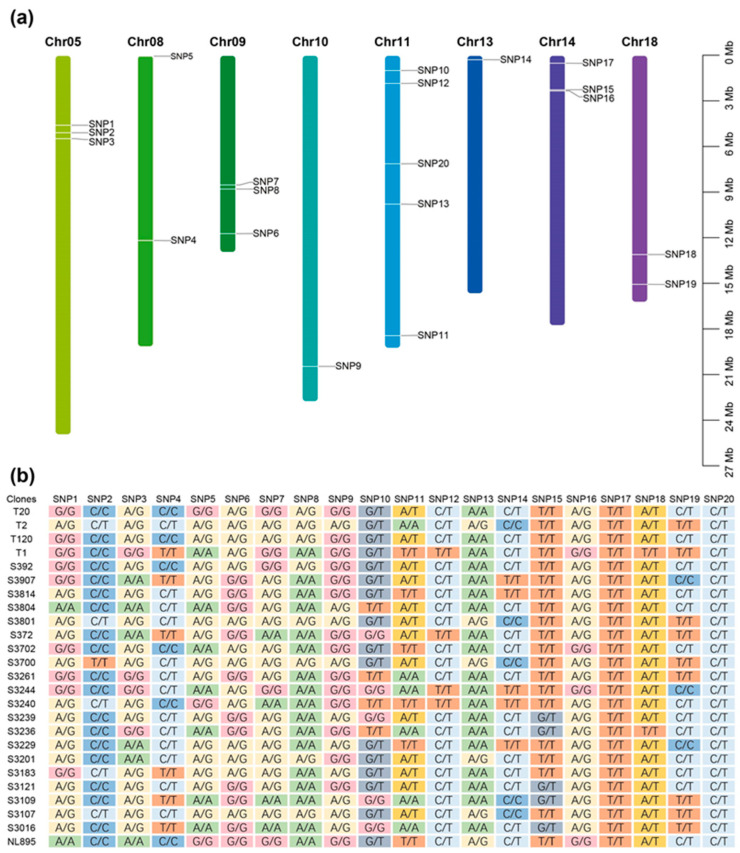
Location and genotype typing of candidate single-nucleotide polymorphisms (SNPs). (**a**) Location of candidate SNPs on the chromosome. (**b**) Genotyping of candidate SNPs. Columns represent different SNPs and rows represent different clones.

All samples were covered by an average of 243.76 reads per SNP locus. The sequencing depth distribution of all samples is shown in Figure S1. The candidate SNPs were distributed across eight chromosomes, with chromosome 11 being the most abundant and chromosomes 10 and 13 being the least abundant, both having only one SNP (Figure 2a). Of the 20 SNP loci measured, SNP17 and SNP20 were not polymorphic in these 24 clones. All SNP genotyping is shown in Figure 2b. With an average of 0.251 (Table S5), the minor allele frequency (MAF) ranged from 0.08 to 0.44. Three markers in total had an MAF below 0.2, indicating that the majority of the markers had high polymorphism.

### 2.3. Selection of Growth Curve Models

**Figure 3:**
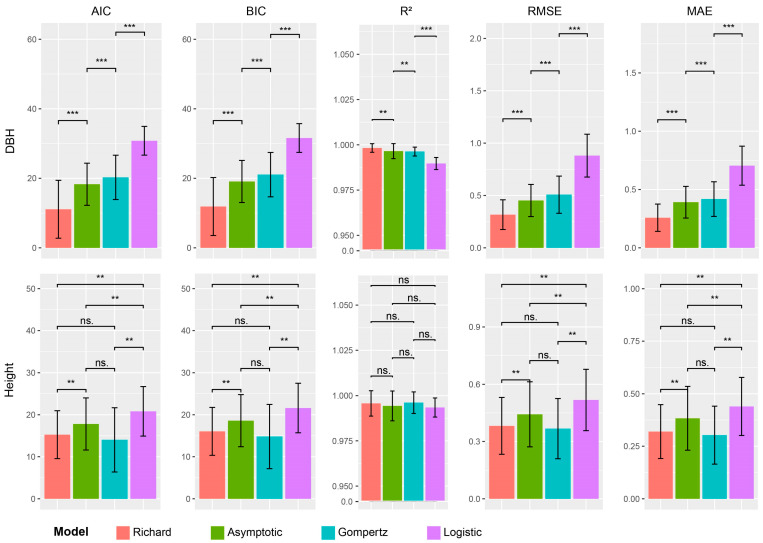
Evaluation parameters of four growth models for diameter at breast height (DBH) and height. The four growth models were Richard, asymptotic, Gompertz, and logistic regression. AIC, Akaike information criterion; BIC, Bayesian information criterion; R^2^, coefficient of determination; RMSE, root mean square error; MAE, mean absolute error. ns., not significant; *p* > 0.05; *, 0.01 < *p* < 0.05; **, 0.001 < *p* < 0.01; ***, *p* < 0.001.

To choose a best-fitting curve model, the growth curve equations of all poplar clones were successfully fitted using the four S-shaped curve models, namely asymptotic, Gompertz, logistic, and Richard. Their performances were assessed using five metrics, including AIC, BIC, R^2^, RMSE, and MAE. The results showed that for the DBH trait, the values of AIC, BIC, RMSE, and MAE for the Richard model were all significantly smaller than those of the remaining three models, while its R^2^ was also significantly higher than the other models; thus, the Richard model had a better goodness of fit for DBH (Figure 3).

For height, there was no significant difference in R^2^ between all four S-sharped models. The AIC, BIC, RMSE, and MAE values for the Richard model were significantly different from those for the asymptotic model and the logistic model but not significantly different from those for the Gompertz model. However, the Gompertz model, which was not significantly different from the Richard model, also did not show significant differentiation from the asymptotic model. Therefore, the best model for height was selected as Richard’s model after multiple comparisons.

**Figure 4:**
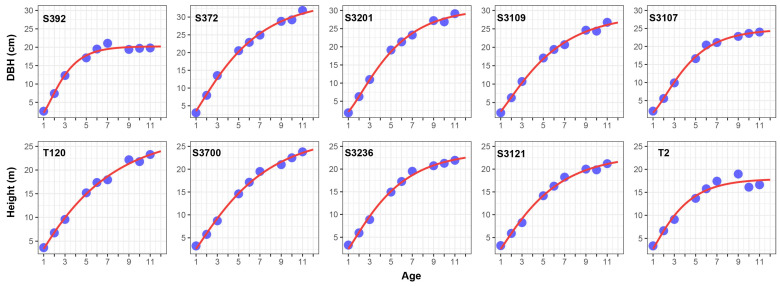
Five samples with the smallest R^2^ (coefficient of determination) of Richard’s model for diameter at breast height (DBH) and height (Dots are actual observed values).

Combining the fitting effects of DBH and height, the Richard model has better performance for both, so the Richard model is selected. Fitting all the samples with Richard’s model (Figure S2), the average fit of DBH and height for all the samples is greater than 0.995, which is an extremely high level (Figure 4 demonstrates the five samples with the lowest R^2^ in DBH and height), which suggests that Richard model is quite robust. In the subsequent association analysis, the parameters were treated as latent variables.

### 2.4. Estimation of Parameters in Richard Growth Model

By analyzing differences between genotypes of SNPs (Table S6), we found that considerable variability was observed in the majority of SNPs, with 10 SNPs with significant differences in DBH and 11 SNPs with significant differences in height. The growth trajectories were fitted in relation to significant SNP loci, as illustrated in Figure S3. It was observed that the significant loci of the same SNP for height and DBH varied widely. For example, SNPs 14, 15, 16, and 19 were found to be significant for DBH but not for height, whereas SNPs 3, 4, 5, 11, and 18 were significant for height but not for DBH. These SNPs predominantly influenced the growth of one trait. However, there also exist some SNPs that are significant for both traits, such as SNPs 1, 7, 8, 10, 12, and 13. This indicates that different SNPs have varying effects on different traits, highlighting the pleiotropy of genes. 

**Figure 5:**
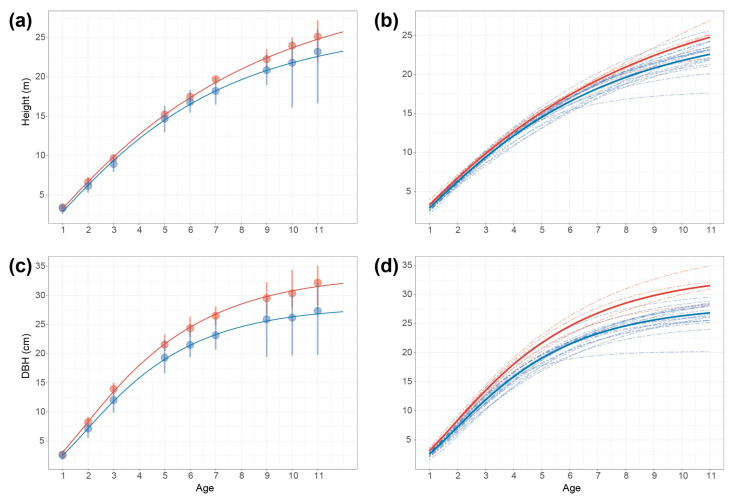
Growth trajectories with different genotypes of SNP12. (**a**,**c**) Growth trajectories of different genotypes of SNP12 for height and diameter at breast height (DBH). Red line is T/T genotype, and blue line is T/C genotype. (**b**,**d**) Growth trajectories of individuals with different genotypes of SNP 12 for height and DBH. Red lines are T/T genotype, and blue lines are T/C genotype. Each individual is marked by a dashed line (T/T genotype in red, T/C genotype in blue).

The method we employed in our study allowed us to decipher the influence of SNPs on growth trajectories effectively. We specifically focused on SNP12, which is highly significant for both DBH and height, to explain its effect on the growth trajectories of these traits. In Figure 5a,c, it can be observed that the growth trajectories of the two genotypes of SNP12 in height were relatively similar before the age of 5 years, but after that point, the differences between the genotypes became more pronounced. For DBH, the growth trajectories of two genotypes exhibited variability from the beginning, and the differences increased with age. To gain a better understanding of the distribution of the two genotypes of SNP12 in clones, we conducted a fitting analysis on all clones containing both genotypes of SNP12 (Figure 5b,d). The results indicated a more distinct differentiation between the two genotypes in clones, with DBH showing better differentiation compared to height.

### 2.5. Determination of Ontogenetic Stages

**Figure 6:**
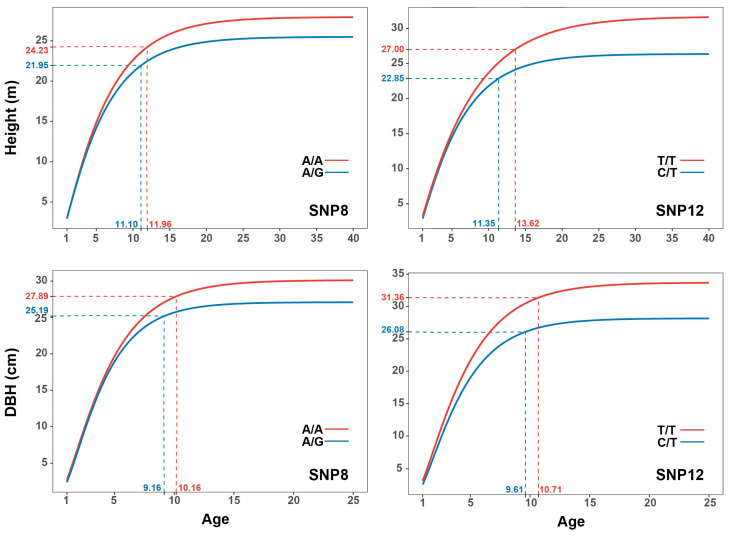
Maximum curvature points and prediction of growth trajectories with different genotypes of SNPs (e.g., SNP8 and SNP12) for height and diameter at breast height (DBH).

**Figure 7:**
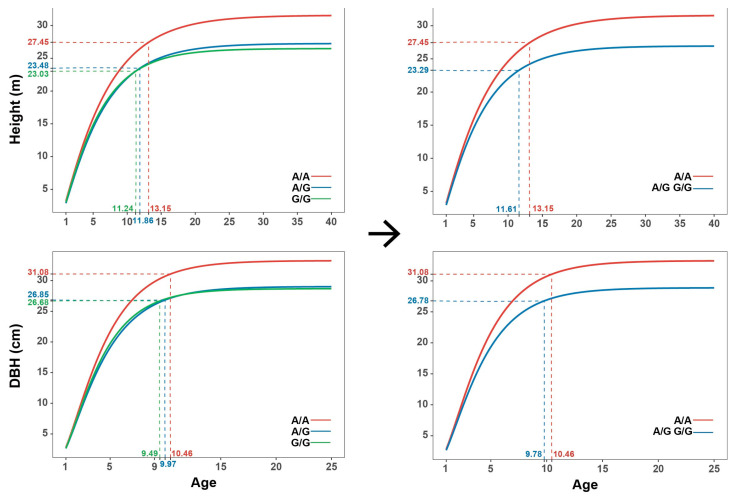
Maximum curvature points and prediction of growth trajectories with different genotypes of SNP1 for height and diameter at breast height (DBH) and combination of genotypes with similar growth trajectories.

Based on the points of maximum curvature in the growth model, two distinct phases of growth were identified: rapid growth and reduced growth (Figure 6). For both DBH and height, the points of maximum curvature predicted that DBH would reach the reduced growth phase before height, with height at 11 to 14 years of age and with DBH at 9 to 11 years of age. This can provide an insight into the rotation age of poplar. Additionally, based on our growth trajectory predictions, DBH reaches its maximum growth earlier than height. Moreover, the time at which DBH and height reach maximum growth varies considerably, with DBH peaking at approximately 25 years of age and height reaching its maximum at approximately 40 years of age. This suggests that DBH and height have different growth patterns and rates of development throughout the growth period. Differences between SNPs in growth trajectories were identified and are displayed in Figure 3. Three SNPs (Figure 6 and Figure 7) with substantial genotypic differences are representatives. Both DBH and height entered the reduced phase later in the better-performing genotypes compared to the other genotypes. Genotypes of SNPs with later time points experienced a prolonged growth period, resulting in greater growth.

The analysis of growth trajectories based on SNP1 genotypes reveals interesting findings regarding the phenotypic differences among the three genotypes, namely G/G, A/G, and A/A. The G/G and A/G genotypes exhibited similar growth trajectories. suggesting that they can be considered as the same genotype in terms of growth patterns. Meanwhile, there is a substantial difference in growth trajectories between these two genotypes and the A/A genotype (Figure 7). When comparing the number of genotypes of SNPs, the proportions of the genotypes of SNPs conform roughly to Mendel’s law of monogenic inheritance.

### 2.6. Effect Modes of Significant SNPs

To further validate the effect of SNPs on DBH and height, we performed association analyses of phenotypes with SNPs for each year using general linear model (GLM) analysis. Through the GLM analysis (Table S7), we were able to identify seven SNPs that exhibited significant associations with growth traits at various ages—six for height and three for DBH. We confined our attention to SNP10 and SNP12, which showed significant correlation with growth and developmental processes in both analyses. In the determination of growth trajectory comparisons across genotypes, they showed significant variability in both DBH and height. Moreover, in determining their associations with time-point traits, SNP10 was significantly associated with height at 3 years and with DBH at 7 years, and SNP12 was significantly associated with height at 2 years and with DBH at 8 years.

Additionally, the model parameters were functionally mapped as multiple traits to the growth trajectory of SNP10 and SNP12 due to the high correlation between the model parameters (Figure S4). SNP12 was identified as significantly associated with the growth trajectory of DBH (*p* < 0.05) (Table S8). Combined with the above analyses, this suggests that SNP10 and SNP12 may be the key loci influencing growth trajectories, with SNP12 having a greater influence. Based on the annotation information (Table S9), we found that SNP10 is located on the *O*-fucosyltransferase gene, and SNP12 is located on the *PCFS4* gene, which encode *O*-fucosyltransferase family protein and PCF11P-similar protein, respectively. *O*-fucosylation plays important roles in various biological processes, including protein–protein interactions, cell signaling, and development [23]. *PCFS4* plays a key role in the promotion of flowering and is involved in mRNA maturation processes [24]. Their functions were related to the physiological activity of life over a long period of time.

## 3. Discussion

Successive years of growth are a distinctive characteristic of forest growth, setting it specifically apart from annual herbs. The stem growth of forest trees is synergistically expressed through the combined interaction of the tree’s inherent genetic characteristics and the surrounding environment. This intricate process involves the coordination and control of numerous minor genes, which work together through a complex genetic mechanism [25]. In the case of perennial poplars, stem growth involves both radial (DBH) and axial (height) growth, with these traits being important indicators of tree growth dynamics. Therefore, annual measurements of height and DBH are useful for further understanding the dynamic growth influenced by internal and external factors. In correlation analysis, when comparing DBH and height, it is observed that height is more influenced by environmental factors. It can be predicted that the heritability of height may be lower compared to DBH, which is similar to previous studies [9]. By fitting growth curves to all individuals, it has been determined that the Richard function best describes the potential growth trajectories of poplar clones. Based on the points of maximum curvature, it has been predicted that poplars would enter a period of reduced growth at certain points in time. Specifically, DBH is predicted to enter a period of reduced growth at 9 to 11 years, while the height is predicted to enter a period of reduced growth at 11 to 14 years under these environmental conditions. The current study also aligns with previous research, such as the study conducted by Sperandio et al. [26], which found that it is economically sustainable to fell poplar at 11 years within a rotation in poplar plantation forests. Furthermore, another study by Niemczyk et al. [27] suggested that the rotation age for felling poplar is greater than 10 years. This study provides valuable insights for predicting and studying the rotation age of poplar trees.

For forest trees, genetic regulation varies at each stage of their long growth cycle, which leads to unstable gene expression at each forest age [28]. Gene loci for growth identified using molecular markers are often limited to specific stages or points in time, which may not provide sufficient reliability for studying multiyear growth changes in forest trees. Few significant SNPs were identified in multiple years, and most of the SNPs exhibited temporal specificity, as previously reported [29,30,31]. These findings also imply that complex growth traits in forest trees may be controlled by a limited number of gene loci that exhibit expression at multiple time points, as well as a larger number of time-specific genes that play specific roles during various developmental stages. The identification of these dynamic gene loci is meaningful and can serve as valuable scientific groundwork for enhancing our understanding of the dynamic growth patterns in forest trees. Using a single point in time overlooks many factors that influence the dynamic stem growth process. We modeled the growth trajectories of each candidate locus with the aim of dissecting the genetic basis of the dynamics of annual growth.

By comparing the growth trajectories, we were able to identify specific SNPs that are associated with the dynamic growth of poplar. The significant SNPs can be annotated to genomic regions of candidate genes that encode specific biological processes (Table S9). For example, SNP1 is located within the *CBS* gene, which predominantly plays a role in RNA metabolic processes and responding to abiotic stress [32]. SNP8 is located within the *PTEN2* gene and is involved in dephosphorylation, response to osmotic stress, and response to salt stress [33]. SNP13 is localized within the *CCP1* gene, which is known to influence the development of floral organs and contribute to the overall developmental process of various growth traits [34]. These SNPs are closely linked to vital biological functions necessary for long-term life activities, implying their potential involvement in the entirety of forest growth and development.

Throughout the growth of poplar, there exists a competitive and cooperative relationship between height and DBH. However, this relationship may vary and sometimes be inconspicuous at different stages of growth. A comparison of growth trajectories and complementary association analyses revealed that SNP12 all showed a significant correlation with the growth of DBH. Observations between SNP12 and various growth traits in poplar strongly suggest that SNP12 plays a critical role as a primary locus in regulating the growth and development of these trees. Further investigations into the *PCFS4* gene, which encodes PCF11P-similar protein 4 and is localized within SNP12, have revealed several noteworthy findings. Firstly, it has been observed that *PCFS4* plays a key role in the promotion of flowering and is involved in mRNA maturation processes [35]. Additionally, *PCFS4* has been associated with heteromorphic leaf variegation, a phenomenon that may play a role in regulating plant adaptation to the environment [24]. In poplar, it has been demonstrated that the *PCFS4* gene exerts a positive regulatory effect on both the main stems and roots of these plants. This pleiotropic influence on the growth of main stems and roots aligns with the results obtained in the present study [36].

In this study, we utilized eleven years of growth data on height and DBH from *P. deltoides* to compare the fit of different models. By fitting four growth models, we ultimately constructed the Richard model as the best-fit model. We divided growth stages by comparing growth trajectories and identified important SNP loci associated with growth trajectories. By identifying the specific loci and determining ontogenetic stages, the study provides valuable insights into the molecular mechanisms that influence variations in longitudinal genetic contributions during stem growth.

## 4. Materials and Methods

### 4.1. Plant Materials and Trait Measurements

In this study, the plant materials used were obtained from the germplasm repository of *P. deltoides* located at Chenwei Forest Farm in Sihong County, Jiangsu Province, China. These plant materials were then cultivated and grown at Siyang Forest Farm in Siyang County, Suqian City, Jiangsu Province, China. This region belongs to the transitional climate of the northern subtropical monsoon with an average annual temperature of around 14.4 °C and an average annual precipitation of about 857.4 mm. The poplar plants comprised twenty-four *P. deltoides* clones and a hybrid poplar NL895 (*P. deltoides* × *P. euramericana* cv. ‘NL895’) as the control genotype. The genotype NL895 was bred from hybridizing the diploid female clone I69 with the diploid male clone I45.

A field trial was performed in a randomized complete block design (RCBD) with three replicates and twenty-five poplar clones in each plot. There was a total of 3–4 ramets per poplar genotype in each plot. Poplar plants were grown at 5 m × 6 m spacing. Height and DBH were measured for each tree over a discontinuous period at tree ages of 1, 2, 3, 5, 6, 7, 9, 10, and 11 years. Height was measured using a hypsometer and recorded with a resolution of 0.1 m, and DBH was measured using Diameter tape (WIN TAPE, China) to measure the diameter of the tree trunk 1.3 m above the ground and recorded with a resolution of 0.1 cm.

### 4.2. DNA Extraction, Resequencing, and Genotyping

The 20 candidate loci used in this study originated from previous studies [19]. These loci have been found to show significant associations with growth traits (Table S10). The target fragment, spanning a region of 400 bp, encompassed 200 bp upstream and downstream of the 20 candidate loci. The primer sequences were designed on both sides of the target sequence using Primer 5.0 primer design software (Table 1).

Fully expanded leaves were collected from each poplar clone and were used for genomic DNA isolation using the Plant Genomic DNA Extraction Kit (DP360) (TIANGEN, Beijing, China). The quality and integrity of isolated DNA were assessed using a NanoDrop-2000 UV spectrophotometer (Thermo Fisher Scientific, Waltham, MA, USA) and 1% agarose gel electrophoresis. The resulting DNA extracts were stored at −20 °C for backup. The polymerase chain reaction (PCR) products were then purified using a Gel Extraction System B Kit (BioDev-Tech, Beijing, China). Subsequently, the purified amplicons from different genotypes were mixed in equimolar concentrations for MiSeq DNA library construction. Finally, the library was sequenced on the Illumina MiSeq platform (Illumina, San Diego, CA, USA) to generate 2 × 250 bp paired-end (PE) reads with an overlap length of 50 bp.

To obtain high-quality clean data, the raw MiSeq reads were trimmed using FASTP (v 0.23.4) [37] under default parameters. The clean reads were then aligned to the reference sequences using bwa-mem2 (v2.2.1) [38]. SNP variants were called on the resulting BAM files using SAMtools (v1.9) [39] and inspected visually with IGV (Integrative genomics viewer, v2.11.0) [40]. SNPs were annotated by SnpEff (v4.2) with the *P. trichocarpa* genome (v4.1) on Phytozome (https://phytozome-next.jgi.doe.gov/, accessed on 24 January 2023).

### 4.3. Model Evaluation

Taking into account the dynamics of growth traits, we employed four distinct non-linear models to fit the growth curves. The equations are expressed as [4]:

(1) $$y=a\times e^{-b\times c^{t}}\;\mathrm{Gompertz}$$

(2) $$y=a\times {\left(1-e^{-b\times t}\right)}^{c}\;\mathrm{Richard}$$

(3) $$y=\frac{Asym}{\left(1+e^{\frac{xmid-t}{scal}}\right)}\;\mathrm{Logistic}$$

(4) $$y=a+\left(b-a\right)\times e^{-e^{c}\times t}\;\mathrm{Asymptotic}$$

where *Asym* and *a* are the maximum value of growth, *xmid* and *c* are closely related to the growth rate, and *scal* and *b* are the characteristic speed of the growth.

The model fitting was performed using the nls function in the R package stats (v4.3.1) [41]. To evaluate the accuracy of the prediction model, statistical measures such as the coefficient of determination (R^2^), Akaike information criterion (AIC) [42], Bayesian information criterion (BIC), root mean square error (RMSE), and mean absolute error (MAE) were used. R^2^ has a range of potential values between 0 and 1, with values closer to 1 indicating a strong correlation between the regression line and the data points. The correlation is higher for the other four metrics, AIC, BIC, RMSE, and MAE, when their values are lower.

### 4.4. Hypothesis Testing

Statistical methods such as maximum likelihood or regression analysis are typically employed in genetic studies to calculate genetic effects and other model parameters. These methods allow researchers to quantify the extent to which genetic factors contribute to observed variations in traits or outcomes. The likelihood ratio (LR) test is a statistical test for comparing the goodness of fit between two statistical models [43]. The form of the likelihood ratio statistic is as follows:

LR = −2(*logL*_0_
− *logL*_1_) (5)

where *L*_0_ represents the maximum likelihood value of the SNP model, while *L*_1_ represents the maximum likelihood value of different genotypes for the SNP. Based on the likelihood values *L*_0_ and *L*_1_, we detect the presence of QTLs that influence the traits height and DBH by comparing the differences in genotype associations through the calculation of LR statistics. The LR statistic approximately follows the chi-square distribution, and its degrees of freedom are determined by the difference in the number of model parameters between *df*_0_ and *df*_1_ (*df*_0_ represents the number of model parameters in *L*_0_, while *df*_1_ represents the number of model parameters in *L*_1_). The chi-square test was used to detect whether there were differences between genotypes of SNPs.

### 4.5. Statistical Analyses

The chi-square test and Student’s *t*-test were performed using the stats package. A *p* value of <0.05 was used to determine statistical significance. The different growth periods were classified by points of maximum curvature [44]. The points of maximum curvature were determined using the maxcurv function in the R package soilphysics (v5.0). Single temporal association mapping was conducted using a general linear model in TASSEL v5.0, and functional mapping [20] through multiple-trait associations was conducted using GMAT [45]. The one-way ANOVA was conducted using R.

## 5. Conclusions

In the study, we performed a longitudinal genetic analysis on the height and DBH of eleven-year-old *P. deltoides* clones using an ultra-deep sequencing dataset. To investigate the genetics of growth, we compared four S-shaped growth models, fitted the growth trajectories of clones, and determined poplar ontogenetic stages by virtue of growth curve fitting and likelihood ratio testing. Additionally, we discovered several significant loci affecting the growth process and demonstrated their effects on the growth trajectories. These results describe the multiyear dynamics of height and DBH in *P. deltoides* and give an insight into the longitudinal trajectory of poplar growth traits.

## Figures and Tables

**Table 1:** Primer sequences for 20 SNP markers.

SNP	Alleles	Forward Primer (5′–3′)	Reverse Primer (5′–3′)	Product Size (bp)
SNP1	A/G	TGGTGAACAGTCAAAGTAACCT	GCATTAAAACTGAGAATGGACA	364
SNP2	C/T	GGCCACAAAATGAATTTCAGAAG	GCTATCACAGTTGCAAATTGTCA	384
SNP3	A/G	GTTTATTGATTGGGTGTTGTTTG	GATGAAATGCGCTAAAAATCC	376
SNP4	C/T	CAACCAAAACTGCTCCACATA	GCAATCAAACTCATCCAATACA	362
SNP5	A/G	AGTCTACACGAGCCCTTCATTT	ATGGAAGCAAGATGGCTAGACT	371
SNP6	A/G	GAAGCACTTGAGGAGCCTTGA	CACCGTGCTTGTCCTGTTTCT	359
SNP7	A/G	GTCCCTGGATTTCAATCTCA	TACGAATCCCATATGTACCTCC	354
SNP8	A/G	TGATATTATCAGCTGTGTTGGGG	TCCTGGTTCTGAAGTTAGCTTCT	364
SNP9	A/G	ATCAACAGGGTGCATAAGAGAA	TTTTATGCTTTTGCTGCCTTC	341
SNP10	G/T	ACTGCTTTAACCAGCTTGTATCCT	CTCAACCCAAATCCACACAATA	380
SNP11	A/T	TTAGGCTGAGAGAACCTCGTG	TTCCTCGTATACCGCTCTCTA	370
SNP12	C/T	TTGTGCACTGTCTTGGAAGGT	CCTTTAGCGGTCAGCAATGTT	388
SNP13	A/G	TACAATGTTCACAAACCTTCC	GAAAAGTTTTCTTTAACATGCTG	346
SNP14	C/T	GGAATTCAAGAACCTGCAATCA	TTCAATTGCTAGAACTCTGGGTC	353
SNP15	G/T	CCAAGTATATCTGACCTAGTTTGC	TAAGAAGCTATGCTGCTCAGTT	348
SNP16	A/G	GAAAGAAGCGTAAGGGCACTG	ACACGATTGGATGAGGCAAG	358
SNP17	C/T	TATGCGGTGTCTCAGACTCTCA	CCGGTATTCTGCTTTGTTTATCC	366
SNP18	A/T	CGCGAGTTGAAATTGAAGAGTC	CATTTTCCCAACAAGCATCGT	362
SNP19	C/T	ATGCATGGTGGCTAGCTAAC	GCCTAGGAAGCTGAACTTGTG	358
SNP20	C/T	TTTGGACTTGATGACTGCAAAC	AAGCTGAATGCATAGCCTGAA	364

## Data Availability

Sequencing data generated in the study are available in the NCBI Sequence Read Archive (SRA) under BioProject accession PRJNA1025043.

## Supplementary Materials

The supporting information can be downloaded at: https://www.mdpi.com/article/10.3390/ijms242216192/s1.
