# Supplementary figures and images for: Analysis of Growth Trajectories and Verification of Related SNPs in Populus deltoides
Source: Int J Mol Sci. 2023 Nov 10;24(22):16192. doi: 10.3390/ijms242216192 (PMC10670923; doi:10.3390/ijms242216192)

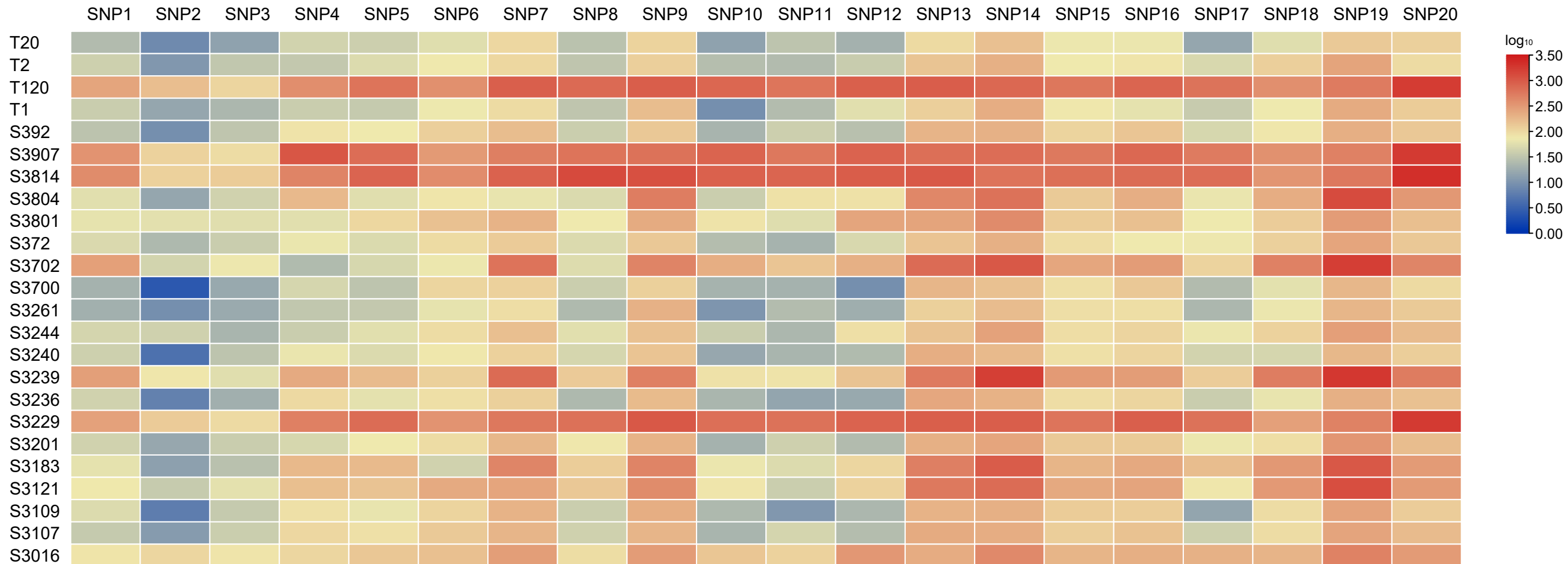

Supplement: Supplementary file 1 [file ijms-24-16192-s001.zip › Figure S1. Heat map of deep-sequencing of 24 clones.pdf]

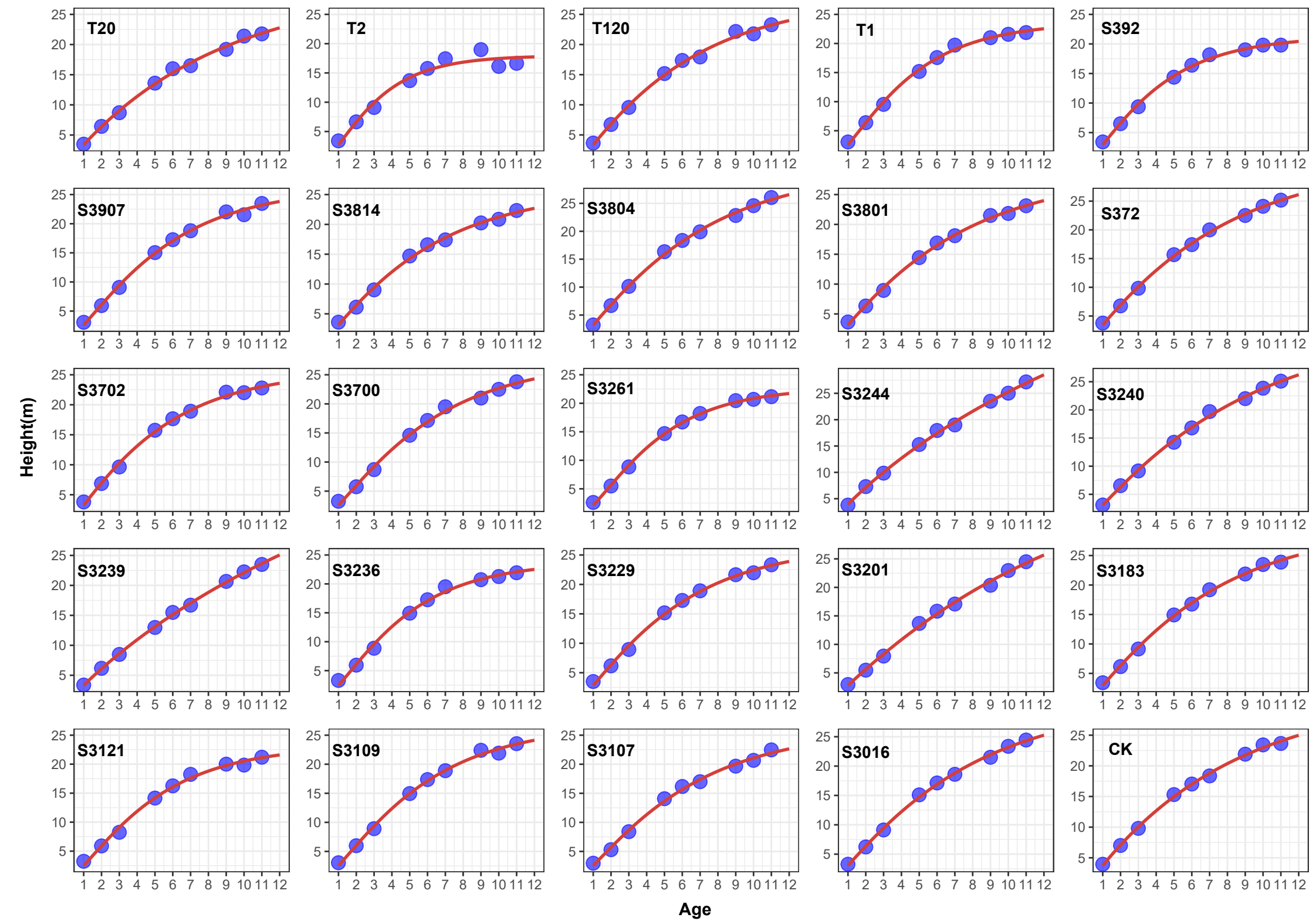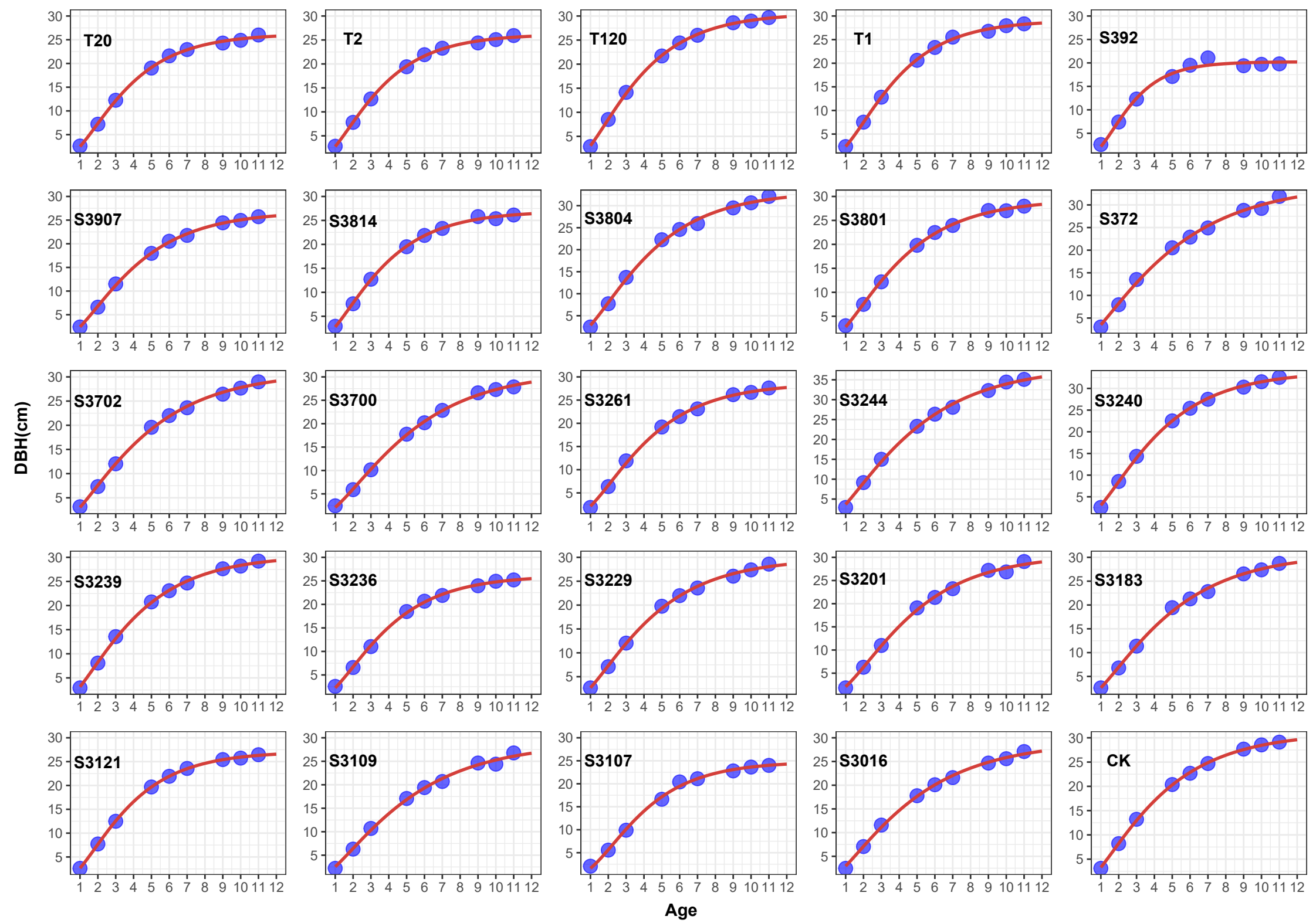

Supplement: Supplementary file 1 [file ijms-24-16192-s001.zip › Figure S2. Richard's model for DBH (diameter at breast height) and Height.pdf]

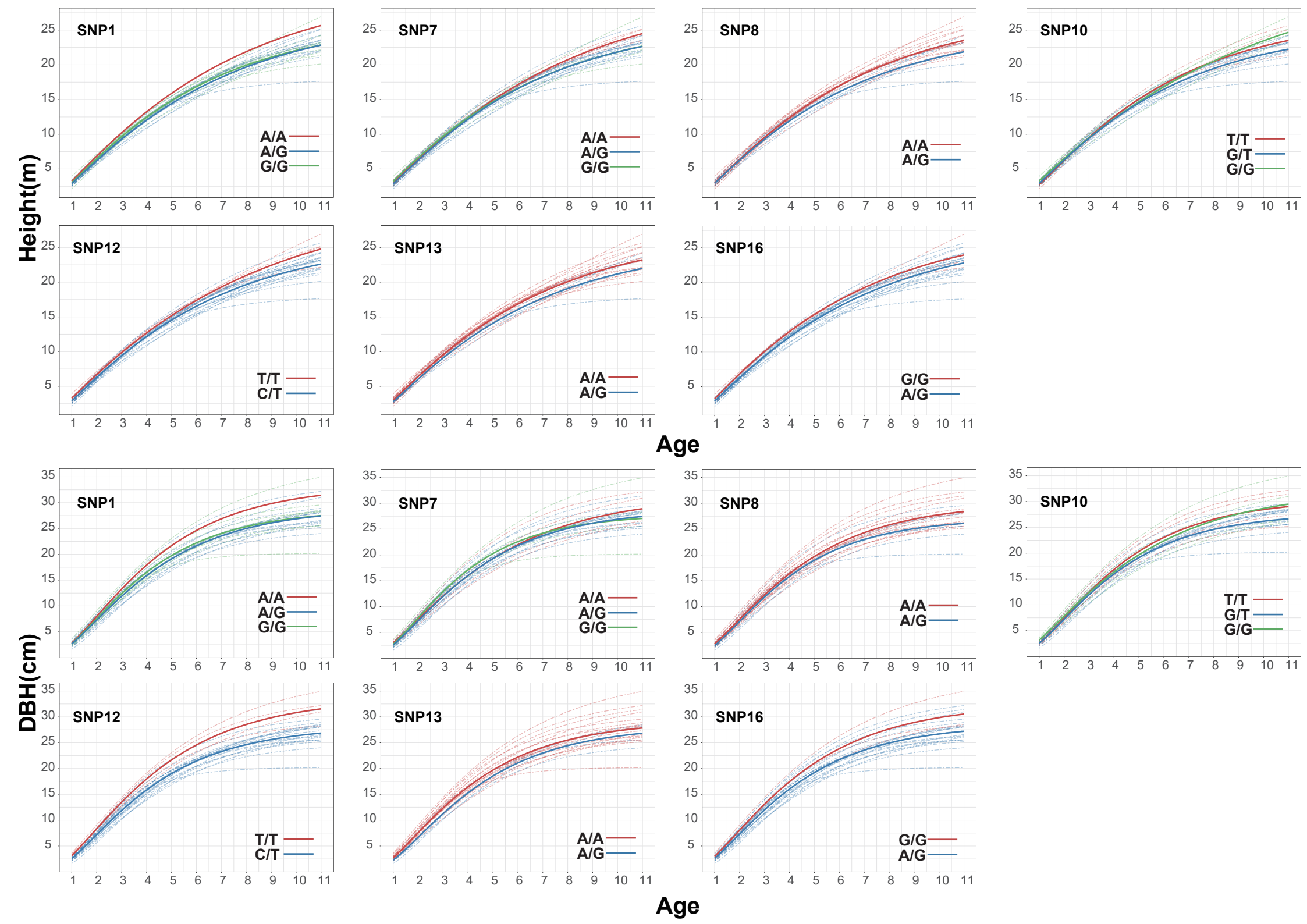

Supplement: Supplementary file 1 [file ijms-24-16192-s001.zip › Figure S3 Growth trajectories with different genotypes of significant SNPs for DBH (diameter at breast height) and Height.pdf]

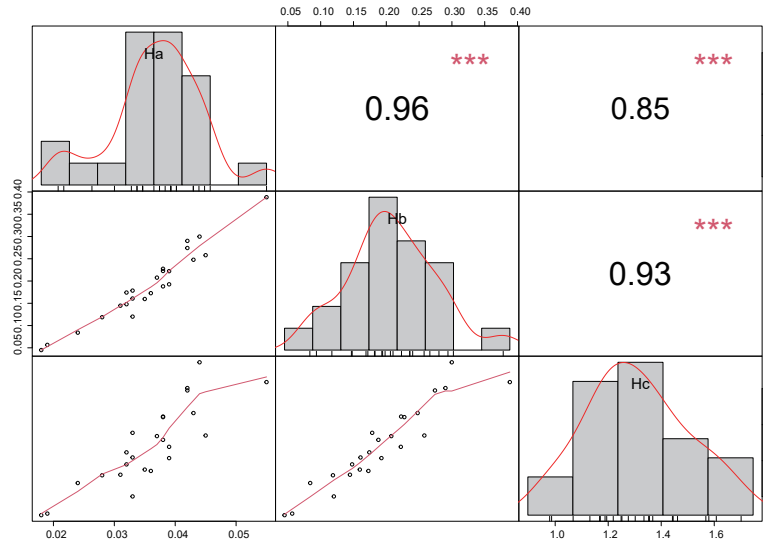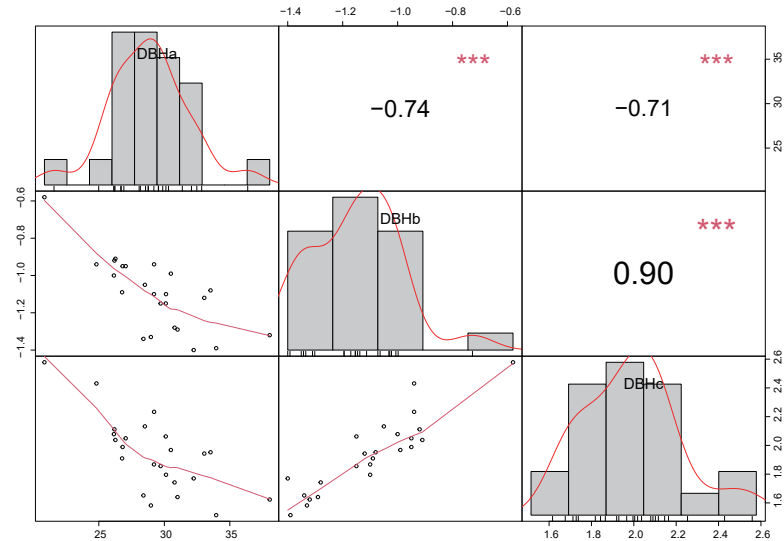

Supplement: Supplementary file 1 [file ijms-24-16192-s001.zip › Figure S4. The Pearson correlation coefficients between model parameters of the fitting curves for H and DBH.pdf]
